# Prevalence and predictors of adverse events following exposure to long-lasting insecticidal nets used for malaria prevention: a community based cross-sectional study in the Democratic Republic of the Congo

**DOI:** 10.1186/s12936-023-04458-w

**Published:** 2023-02-01

**Authors:** Gillon Ilombe, Thérèse Mpiempie, Gauthier Mesia, Junior R. Matangila, Aimée M. Lulebo, Vivi Maketa, Baby Mabanzila, Nicole M. Muela, Flory T. Muanda, Sylvie Linsuke, Jean-Pierre van Geertruyden, Pascal Lutumba

**Affiliations:** 1 Unit of Entomology, Department of Parasitology, National Institute of Biomedical Research, Kinshasa, Democratic Republic of the Congo; 2 Unit of Clinical Pharmacology and Pharmacovigilance, Department of Base Science, Faculty of Medicine, University of Kinshasa, Kinshasa, Democratic Republic of the Congo; 3 Faculty of Medicine, Global Health Institute, Antwerp University, Antwerp, Belgium; 4 Department of Tropical Medicine, Faculty of Medicine, University of Kinshasa, Kinshasa, Democratic Republic of the Congo; 5 Faculty of Medicine, Public Health School, University of Kinshasa, Kinshasa, Democratic Republic of the Congo; 6 Expanded on Inoculation Logistic Section, Ministry of Public Health Kinshasa, Kinshasa, Democratic Republic of the Congo; 7 ICES, Toronto, ON Canada; 8 Department of Physiology and Pharmacology, Western University, London, Ontario Canada; 9 Department of Epidemiology, National Institute of Biomedical Research, Kinshasa, Democratic Republic of the Congo

## Abstract

**Background:**

Malaria morbidity and mortality increase in the Democratic Republic of the Congo (DRC) may be the consequence of the low utilization rate of long-lasting insecticidal nets (LLINs) resulting from poor compliance due to adverse events (AEs). This study aimed at determining the prevalence and predictors of AEs following the mass distribution of LLINs in the Kisantu Health Zone (KHZ), a high malaria-endemic region in the DRC.

**Methods:**

A community-based cross-sectional study embedded was conducted within a randomized controlled trial (RCT) after the mass distribution of LLINs in 30 villages located in DRC KHZ. A three-stage sampling method was used without replacement to select 1790 children. Data was collected on adverse events (AEs) using a reporting form and information on demographics, nutritional status, and house characteristics. This was done using a structured questionnaire administered to household heads. Logistic regression models were used to identify predictors of AEs following the mass distribution of LLINs.

**Result:**

In a total of 1790 children enrolled, 17.8% (95% CI 16.1–19.7) experienced AEs. The most common AEs were respiratory-related (61%). Around 60% of AEs occurred within 24 h of use, and 51% were resolved without treatment. Sleeping under deltamethrin LLINs (Adjusted OR, 95% CI 5.5 [3.8–8.0]) and zinc roofing (Adjusted OR, 95% CI 1.98 [1.1–3.57]) were associated with the risk of reporting an AE following the mass distribution of LLINs.

**Conclusion:**

Approximately 1 out of 5 children had an AE within 24 h following LLIN use. These adverse events were often respiratory-related. LLINs and roofing types were associated with a higher risk of reporting AEs. However, further research using a robust study design is needed to confirm these findings. Future studies should design and implement interventions aiming to reduce AEs and improve compliance with LLINs.

## Background

Malaria is a major public health problem in Sub-Saharan Africa. In 2020, the World Health Organization (WHO) reported 627,000 malaria-related deaths, mainly in children under 5 years of age [1, 2]. Nigeria and the Democratic Republic of the Congo (DRC) alone accounted for more than 35% of global deaths due to malaria [3]. Given this heavy burden, in 2000, at the Abuja summit, African countries endorsed the project to roll back malaria in Africa [4]. A statement that at least 60% of people at risk, especially pregnant women and children under 5 years [5], should benefit from the most appropriate therapeutic combination in 2005, and to the implementation of prevention strategies including personal and community protection through the distribution of insecticide-treated mosquito nets and preventive intermittent treatment in pregnant women [6]. In less than a quarter of a century, the impregnated mosquito net has become a cornerstone in malaria prevention in Africa [5]. Approximately 582 million of long-lasting insecticidal nets (LLINs) have been delivered worldwide, including 505 million in Africa between 2014 and 2016 [2]. LLINs are treated with insecticide, which explains why they must be registered like any other medication according to the national legistaltion [6]. Deltamethrin is one of the most pyrethroid insecticides (pesticides formulations) used in the control of malarial vectors, particularly *Anopheles gambiae* [7]*,* while piperonyl butoxide (PBO) is used as a synergist component of pesticide formulations with no pesticidal activity [8].

These insecticides can, in some cases, be responsible for some inconvenience both in humans and in the environment [9, 10]. There were a large number of nets that are prequalified. In 2017, the WHO gave pyrethroid-piperonyl butoxide (PBO) nets an interim endorsement as a new vector control class and recommended that countries consider deploying these nets in areas where pyrethroid resistance to the main malaria vectors has been confirmed [11].

According to the Democratic Republic of the Congo (DRC) National Malaria Control Programme (NMCP) activity reports for 2015 and 2016, a considerable increase in the distribution of LLINs was observed during the previous three years [12]. However, the same reports showed an increase in malaria mortality, especially in children under 5 years of age [3]. While it is not possible to rule out methodological flaws in the data collection and data analysis, two hypotheses have been proposed to explain this paradox: (i) the rise of vector resistance to some insecticides of the pyrethroid family, and (ii) a low utilization rate of LLINs as a consequence of poor compliance due to false perceptions in communities and negative impact of misuses. Data on the safety of LLINs exposure are limited. No study has determined the prevalence and predictors of AEs after exposure to LLINs in the DRC. Given that the distribution of LLINs will increase as part of malaria prevention strategies, it is urgent to determine the prevalence and predictors of AEs in Kisantu, a high malaria-endemic region in the DRC.

## Methods

### Study setting

This study was conducted in Kisantu Health Zone (KHZ), located in Kongo Central Province, DRC, where malaria is highly endemic and with vector populations resistant to pyrethroids. In 2017, the prevalence of malaria was estimated at 36% [13]. Also, the susceptibility testing conducted in the neighbor site, Kimpese, in the same province in 2016 showed that *An. gambiae *sensu lato (*s.l*.) were resistant to permethrin and deltamethrin [12, 13]. In addition, Kisantu is situated in the territory of Madimba, district of the Lukaya, which covers a surface area of 2400 Km^2^ and a population of 153,188. In this area, agriculture is the main activity.

### Study design

A community based cross-sectional study was conducted to analyse safety data collected in the context of an open randomized controlled trial (RCT) evaluating the effectiveness of bed nets treated with deltamethrin + PBO LLINs *vs* deltamethrin LLINs in households over 12 months in the KZH, an area of stable malaria transmission. A three-stage sampling method was used without replacement as follows: within KHZ, in the first stage, four of the 11 health areas having the highest malaria prevalence, based on 2017 parasitological surveys [14], were selected. In the second stage, 30 villages within each of the four health areas were randomly selected. In the third stage, systematic random sampling was used to identify 56 to 60 children aged 0 to10. Finally, this trial enrolled 1790 children, aged 0 to 10, in 30 villages from which 15 were randomly assigned to receive deltamethrin + PBO LLINs (refers as the intervention arm) and the 15 others received deltamethrin LLINs (the control arm).

The intervention brand was PermaNet 3.0, which contained two active ingredients with the following specification: (1) Deltamethrin had a concentration of 4 g/kg for the roof and 2.8 g/kg and 2.1 g/kg for the side, respectively [15]. (2) PBO had a concentration of 25 g/kg for the roof. Deltamethrin is one of the most pyrethroid insecticides (pesticide formulations) used in the control of malarial vectors, particularly *An. gambiae* [7], while PBO is used as a synergist component of pesticide formulations with no pesticidal activity [8]. Before the distribution of mosquito nets in households, a total of 10 mosquito nets randomly chosen in each type were withdrawn and stored at NIRB for the residual determination of delthamethrin.

The control brand was DawaPlus^®^ 2.0, which contained deltamethrin at a 2.66 g/kg concentration for 75-denier yarn and 2 g/kg for 100-denier yarn, corresponding to 80 mg/sq m of deltamethrin, using a polymer as a binder [16]. The details of this trial have been fully described in Clinical Trial.gov (NCT03289663).

After the mass distribution of the LLIN within the 30 villages included in the trial, the households were contacted to determine the prevalence of AEs following exposure to any type of LLIN. Eligible households were those having one or more children below the age of 10, where the household head demonstrated ability and willingness to participate in the trial by signing an informed consent (or fingerprint) and with the assistance of an impartial witness (if the head of the household is illiterate).

### Data collection

Data on adverse events (AEs) was actively collected using a reporting form that a team member completed during a household visit 60 days after the mass distribution of the LLINs. The reporting form contained information regarding (1) the type and the outcome of AEs (date and place of adverse reaction, circumstances of occurrence/description of facts, clinical consequences found, and preventive measures and actions taken) and (2) the medical device used (model, type, impregnation, trade name).

AEs were defined as any harmful and unwanted reaction in any part of the body and were coded using the Medical Dictionary for Regulatory Activities (MedDRA). Blinded physicians trained in pharmacovigilance assessed the causality of AEs according to the following toxicovigilance process [17]: an *imputable case* was defined as a patient who has been exposed to a pesticide and whose clinical and/or biological signs are related to this exposure. An *excluded case* was defined as a patient who has been exposed to a pesticide and whose clinical and/or biological signs are not related to this exposure or a patient who has been exposed to a pesticide but who has not presented any clinical and/or biological sign, or patient who has not been exposed to a pesticide (coding error or erroneous report). A *severe case* was defined as the appearance of a health event that may be linked to exposure to plant protection or antiparasitic product, excluding adverse reactions and having led to hospitalization with a level 3 of the Poisoning Severity Score (PSS) [3], immediate life-threatening, permanent or temporary functional incapacity, disability, congenital anomaly or malformation or death. When the toxicologist did not have enough information (on the product involved and/or the clinical signs) to comment, a patient was classified in an unspecified group.

Data on potential predictors of AE, including demographics, children, and household characteristics, were also collected using a structured study questionnaire during the household visit. These variables were collected during the baseline study (bed net distribution), this choice is justified by the facts that the populations which received the different kind of bed net were statistically different with regards to household and children characteristics. To control the potential confounding factors; these variables have been included in the study. The following variables were included from the base line:

For household characteristics: type of floor, type of wall, type of roofing.

For children characteristics and nutritional status: gender, age, weight, height. Three nutritional status indices were calculated, using Anthroplus application, notably stunting (height/age), wasting (weight/height) and underweight (weight/age) were defined as per WHO child growth standards. Children with Z scores less than two standard deviations (≤ 2 SD) below the median of the WHO child growth standards were classified as malnourished (stunted, wasted or underweight), minus 3 SD indicates severe stunting, wasting, and underweight. Z scores of less than − 2 SD were considered indicative of stunting. Anthroplus application was used to estimate these nutritional indicators.

From the twenty samples stored at NIRB, five pieces per mosquito net was cut off of each mosquito net. This implies that there were 50 pieces per type of mosquito net giving a total of 100 pieces sent to CDC/Atlanta for residual insecticide assay by X-ray Fluorescence Analysis (XRF).

### Statistical analysis

After the survey, a database was created with Epi-info version 7. The data was previously cleaned to identify outliers, and corrections were made when possible. The analyses was performed using Epi-info7 and STATA 13.0 softwares. Descriptive analyses were performed to compare the proportions using the *χ*^2^ test and the Fisher exact test when the *χ*^2^ test could not be applied. Logistic regression was used to identify factors independently associated with AEs and estimated the adjusted odds ratio (OR) and corresponding 95% confidence interval (CI) to quantify the strength and statistical significance of the association. Two-sided p-values of less than 0.05 were interpreted as statistically significant. All independent variables which had a p value < 0.10 in bivariate analysis were introduced in the model using the entry method.

## Results

From March 15 to April 15, 2018, among a total of 1790 children residing in 30 villages in KZH, 48.6% (870/1790) were female, 51.4% (920/1790) were less than five years old, and 49.8%(862/1790) were sleeping under deltamethrin + PBO LLINs. Baseline characteristics are presented in Table 1. DawaPlus^®^, PermaNet 3.0 walls, and PermaNet 3.0 roofs contain 77.4% (95% CI 68.7–87), 78.7% (95% CI 44.6–92.5), and 135.8% (95% CI 125–145.9) mg/m^2^ deltamethrin, respectively (Table 1).

**Table 1:** Socioeconomic characteristic of children according to type of LLINs in Kisantu Health Zone

Variables	Type of mosquito net		p-value
	Deltamethrin LLINs (%)	Deltamethrin + PBO LLINs (%)	
No.	898	892	
Gender			0.959
Female	437(48.7)	433(48.5)	
Male	461(51.3)	459(51.5)	
Age			0.023
≥ 5 years	458(51)	407(48.3)	
˂ 5 years	440(49)	485(54.4)	
Type of floor			0.000
Cement/square	577(64.3)	110(12.3)	
Other floor	321(35.8)	782(87.7)	
Type of wall			0.000
Brick cement or burning	865(96.3)	528(59.2)	
Clay with pillier	33(3.7)	364(40.1)	
Type of roofing			0.000
Zinc	570(63.5)	221(24.8)	
Straw	316(35.2)	425(47.6)	
Stalk/woods	12(1.3)	246(27.6)	
Stunting			0.604
No	541(63.4)	541(64.6)	
Yes	312(36.6)	296(34.4)	
Underweight			0.371
No	675(75.2)	654(73.3)	
Yes	223(24.8)	238(26.7)	
Wasting			0.953
No	764(85.1)	758(84.9)	
Yes	134(14.9)	134(1)	
Chemistry bed net			
Wall	77.4% (95% CI 68.7–87)	78.7% (95% CI 44.6–92.5)	
Roof		135.8% (95% CI 125–145.9)	

Of 1790 children included in the study, 319 (17.8%) experienced at least one AE (Table 2). This implies that for every 5 children sleeping under LLIN, approximately 1 had an AE. Of those who experienced AEs, 17.8% (319/1780) were imputable (Table 2). For every 5 children who experienced an AE after sleeping under a LLIN, approximately 165 (61.1%) had a respiratory adverse event (Table 3). More than half of the AEs (59.9%) had occurred within 24 h of use (Table 3). Of those who had developed AEs in this period, about 4% stopped using LLINs, and 39% of them decided to wash the bednet before any other use. Overall, 51% of AEs were resolved without any treatment, 11.6% used medical treatment and 4.4% used traditional medicine (Table 3). Cough 34.8% (224/643) was the most reported AE (Table 4). Of 319 children who had an AE, 206 (64.6%) had experienced at least one side effect affecting two different systems simultaneously (Table 5).

**Table 2:** Characteristic of children according to the reporting of adverse events post use of the LLIN in Kisantu Health Zone

Variables	Adverse Event		p-value
	No (%)	Yes (%)	
No.	1471	319	
Gender			0.723
Female	712(48.4)	158(49.5)	
Male	758(51.3)	161(50.5)	
Age			0.038
≥ 5 years	694(47.2)	171(53.6)	
˂ 5 years	776(52.8)	148(46.4)	
Type of floor			< 0.001
Cement/square	520(35.4)	167(52.4)	
Other type	950(64.6)	152(47.6)	
Type of wall			< 0.001
Cement brick/cuite burning	1114(75.8)	278(87.2)	
Clay with pillier	356(24.2)	41(12.9)	
Type of roofing			0.002
Zinc	628(42.7)	163(51.1)	
Straw	613(41.7)	127(39.8)	
Stalk/woods	229(15.6)	29(9.1)	
Stunting			0.734
No	880(63.8)	201(64.8)	
Yes	499(36.2)	109(35.2)	
Underweight			0.413
No	1097(74.6)	231(72.4)	
Yes	373(25.4)	88(27.6)	
Wasting			0.367
No	1255(85.4)	266(83.4)	
Yes	215(14.6)	53(16.6)

**Table 3:** Report of adverse events after the use of mosquito nets in Kisantu Health Zone

Variables	Type of mosquito net		All (%)	p-value
	Deltamethrin LLINs (%)	Deltamethrin + PBO LLINs (%)		
Time of occurrence AEs				
24 h	136(53.9)	55(82.1)	191(59.9)	
48 h	95(37.1)	11(16.4)	106(33.2)	
72 h	20(9.6)	2(2.9)	22(6.9)	
Attitude facing at AEs				0.001
Nothing	136(53.9)	26(38.8)	162(50.8)	
Medical Treatment	17(6.8)	20(29.9)	37(11.6)	
Abandonment of LLIN	10(3.9)	1(1.5)	11(3.5)	
Washing of LLIN	89(35.3)	6(8.9)	95(29.8)	
Traditional treatment	0(0.0)	14(20.9)	14(4.4)	
System reached by AEs				0.001
Respiratory	162(64.3)	33(49.3)	195(61.1)	
Cutaneous	17(15.1)	7(10.5)	45(14.1)	
Ocular	10(0.8)	1(1.5)	3(0.9)	
Respiratory + Cutaneous	9(3.6)	26(38.8)	35(10.9)	
Cutaneous + Ocular	41(16.3)	0(0.0)	41(12.9)

**Table 4:** Distribution of adverse events types according to the model of mosquito nets in Kisantu Health Zone

Adverse Events	Deltamethrin LLINs (%) n = 503	Deltamethrin + PBO LLINs (%) n = 140	P
Asphyxia	8(1.6)	5(3.6)	0.169
Asthma	0(0.0)	1(0.7)	0.217
Cough	172(34.2)	52(37.1)	0.518
Dermatitis	19(3.8)	0(0.0)	0.019
Dermatitis bullous	0(0.0)	1(0.71)	0.217
Eye pruritus	6(1.19)	0(0.0)	0.348
Headache	3(0.6)	0(0.0)	1
Lipsoedema	0(0.0)	1(0.71)	0.217
Nasal congestion	5(0.99)	9(6.43)	˂ 0.001
Ocular hypereraemia	35(6.96)	0	˂ 0.001
Pruritus	35(6.96)	5(3.57)	0.204
Rash	8(1.59)	25(17.86)	˂ 0.001
Skin burning sensation	95(18.9)	7(5)	˂ 0.001
Rhinorrhoea	116(23.6)	31(22.14)	0.908

**Table 5:** Undesirable effects number of post-use LLINs to the children in Kisantu Health Zone

Number of AE	Type of mosquito net		All (%)	p-value
	Deltamethrin LLINs (%)	Deltamethrin + PBO LLINs (%)		
1	42(16.73)	13(19.12)	55(17.24)	0.198
2	166(66.14)	40(58.82)	206(64.58)	
3	43(17.13)	14(20.59)	57(17.87)	
4	0	1(1.47)	1(0.31)	

Sleeping under deltamethrin LLINs (Adjusted OR, 95% CI 5.5 [3.8–8.0]) and zinc roofing (Adjusted OR, 95% CI 1.98 [1.1–3.57]) emerged as independent predictors of reporting of AEs following the mass distribution of the LLINs (Table 6).

**Table 6:** Predictors of adverse events post use of LLINs in Kisantu Health Zone

Variables	Univariate analysis Crude OR (95% CI)	p value	Multivariate analysis Adjusted OR (95% CI)	p value
Wall type				
Cement block	2.1(1.5–2.8)	** < 0.001**	1.02(0.66–1.52)	0.916
Mud walls/Straw	1			
Floor type				
Cement/tiles	2.0 (1.5–2.5)	** < 0.001**	1.2 (0.83–1.78)	0.322
Sand/wood	1			
Roofing type				
Zinc	2.1(1.3–3.1)	** < 0.001**	1.98 (1.1–3.57)	0.022
Straw	1.3 (0.9–1.6)	**0.086**	1.39 (0.96–2.02)	0.084
Thatch/wood	1			
Type LLIN				
Deltamethrin	4.8 (3.6–6.4)	** < 0.001**	5.5 (3.8–8.0)	** < 0.001**
Deltamethrin + PBO	1			
Age				
< 5 years	0,7 (0.6–0.9)	**0.038**	0.82 (0.64–1.1)	0.132
≥ 5 years	1			
Gender				
Female	1.04 (0.8–1.3)	**0.723**	0.96 (0.75–1.24)	0.763
Male	1			

Values in bold, means that the p is significant

## Discussion

This study showed that approximately 1 in 5 children had an AE within 24 h following LLINs use. This result was consistent with studies done in Mali demonstrating adverse events after using the LLINs [17]. In addition, the time frame to an adverse event observed in this study was in line with other studies [18]. Of children who experienced AEs, 3 in 5 reported respiratory-related adverse events. This finding differed from Mali's studies because more adverse events were found in the present study. A potential explanation of this discrepancy may be due to environmental, nutritional, seasonality, and genetic differences between populations and data collection differences between the studies. Overall, the nature, severity, and scope of AEs were expected, often resolved naturally, and consistent with earlier studies [19].

Sleeping under deltamethrin LLINs was associated with a five-fold higher risk of experiencing AEs after the mass distribution of LLINs. However, residual confounding factors may partly explain these findings. Deltamethrin LLINs may be less effective at preventing malaria than deltamethrin + PBO. As such, the higher proportion of AEs observed in children sleeping under deltamethrin LLINs may be due to untreated malaria. Several factors could explain this difference in the occurrence of adverse events, including the quality of mosquito nets. In this study, some nets had an incorrect residual dosage, either above or below the manufacturer's declared threshold. Although this study was descriptive and was not designed to compare the risk of reporting adverse events between the two types of mosquito nets, these results warrant further consideration in future studies. Zinc roofing was associated with almost a two-fold higher risk of AEs. These results were unexpected but may warrant further consideration in future studies.

The present study has several strengths. First, this is the first community-based cross-sectional study to determine the prevalence and predictors of AEs after exposure to LLINs in the DRC. Second, this is the first study that captures AEs following exposure to LLINs using active pharmacovigilance surveillance. Third, findings from this study will help design and implement knowledge-based intervention to reduce AEs and improve compliance to malaria prevention strategies in the DRC. Finally, these findings provide hypothesis-generating research to be tested in future studies using robust design. For example, future research should compare the two LLINs safety to determine which option is associated with fewer AEs.

This study has several limitations. First, misclassification of the outcome in this study cannot be ruled out since the AEs were subjectively assessed in a recent survey after the occurrence of the outcome. Second, underreporting is a concern inherent to pharmacovigilance studies. For example, several studies have suggested that less than 10% of detected adverse drug reactions are effectively reported to medicine regulatory authorities [20, 21]. Fourth, it is challenging to derive causal relationships from cross-sectional analysis because of temporality issues [20]. This study was conducted to estimate the prevalence and predictors of AEs, which will help design a comparative safety study of LLINs using a cohort design with appropriate statistical techniques to control confounding factors by indication. furthermore, only some characteristics collected during the base line study were used as predictors. Finally, data on serum deltamethrin and PBO levels were not available, which precluded any corroboration in this study that excessive serum concentrations were the cause for AEs.

## Conclusion

Approximately 1 in 5 children had an AE within 24 h following LLIN use. These adverse events are often respiratory-related. AEs were associated with LLINs and roofing types. In view of the results, delthamethrin in combination with PBO would be the best indicated. However, further research using a robust study design is needed to confirm these findings. Future studies should design and implement interventions aiming to reduce AEs and improve compliance with LLIN.

## Data Availability

All relevant data supporting the findings of this study are included within the paper and their additional files.

## Abbreviations

DRC: The Democratic Republic of the Congo
LLINs: Long-lasting insecticidal nets
IRS: Indoor residual spraying
NMCP: National Malaria Control Programme
PBO: Piperonyl butoxide
HZ: Health zone
KC: Kongo Central
TBS: Thick blood smear
HLC: Human landing catches
SR: Sporozoite rate
HBR: Human biting rate
EIR: Nightly entomological inoculation rate
